# Naval Hospital of St. Petersburg

**Published:** 1829-11

**Authors:** 


### NAVAL HOSPITAL OF ST. PETERSBURG.

*Case of a Tumor of the Radial or Spiral Nerve of the right Arm, removed by* HARRY LEEKE GIBBS, M.D. Member of the Royal College of Surgeons of London.

PHILIP CHILAIEFF, æt. forty-two, a sailor of robust make and plethoric habit, was admitted into the General Naval Hospital of St. Petersburg, on the 24th December, 1828. He stated that, about fifteen years ago, he received a blow with a handspike on the outside of the right arm; that the pain at the time was severe, and followed by numbness down the forearm to the back of the hand and fingers; and that these morbid sensations did not completely subside till some months had elapsed. Half a year from the infliction of the injury, he first perceived a moveable subcutaneous swelling, of the size of a kidneybean, about four inches above the elbow-joint, just below the insertion of the deltoid muscle, painful to the touch, and accompanied by lancinating pain in the course of the branches of the radial nerve, on being pressed. These symptoms were increased by violent exercise or changes of the weather. For twelve years and a half the tumor remained stationary and without pain, when two years back (from long-continued hard labour, as he supposes,) it gradually increased, and at the same time the lancinating pains down the forearm recurred.

At his admission into the Naval Hospital, I discovered a prominent tumor, of the size of a small hen's egg, tolerably moveable, but connected by a cord both above and below, highly painful on being handled, very slightly compressible, and attended by an increase of the symptoms before mentioned. As the patient appeared anxious and excited, I ordered him to lose eighteen ounces of blood, and a smart cathartic to be given. He was put on a very spare diet, and the bowels kept in a lax state.

January 5, 1829, the arm being laid on a table, I made an incision of five inches, extending from the lower part of the deltoid to within an inch of the outer condyle of the os brachii. On dissecting back the integuments, a tumor of a bluish white colour was brought into view. I found it occupying the fossa, formed by the insertion of the deltoid muscle above, the triceps extensor cubiti behind, the origin of the supinator radii longus below, and the brachialis externus within. Under the outer portion of the latter muscle part of the tumor had imbedded and attached itself, so that it became necessary to remove it. Having separated the tumor from its lateral attachments, I divided the nerve three quarters of an inch below it. The man instantly exclaimed that he had lost the power of raising his fingers, as well as the feeling in the outer part of the forearm and back of the hand. The dissection beneath was now easy, and I was happy to find that my fears of an adhesion to the periosteum were groundless. I lastly cut through the nerve at the like distance above the tumor, just as it emerges from beneath the bone. This was attended with pain, and as the artery accompanying the nerve bled freely, a ligature was applied. The vessel seemed dilated, and to be the arteria nutriens of the tumor. The wound was brought together by strips of adhesive plaster, over which graduated compresses and a circular bandage were applied. The limb was placed on a pillow in a relaxed position, the forearm and hand enveloped in flannel, and a bottle of warm water ordered to be constantly applied to the fingers.

January 4, second day.—He passed but a restless night, notwithstanding the administration of two grains of opium yesterday evening. He complains of oppression under the sternum, and a sense of constriction across the lower part of the chest and præcordia; also of pains shooting up the right side of the neck, in the course of the cervical nerves forming the brachial plexus. As the patient appeared agitated and the pulse rising, two pounds of blood were directed to be drawn from a large orifice, and an active purge was prescribed. By the evening copious evacuations had taken place, and he was composed.

Third day.—He slept tolerably; the pain was more concentrated to the left side of the thorax and right hypochondrium. A large blister was applied over these parts.

Fourth day.—Much better. The strictest antiphlogistic plan was followed, and the bowels kept well open by a saline mixture with the sulphate of magnesia.

Fifth day.—About half the wound was found healed by the first intention. He now complains chiefly of an acute pain at the extremity of the right thumb-nail, especially on its being touched. He has no feeling on the radial and outer side of the forearm, back of the thumb, and those fingers supplied by the dorsal branch of the radial nerve. It is curious that a well-defined limit exists, extending as far as the middle of the ring finger; for the inside of this and the whole of the little finger, supplied by the ulnar nerve, are sensible on being pinched.

Sixth day.—The wound is suppurating freely at the lower part.

Ninth day.—The ligature came away. The patient sat up for a few hours. The wound is filling up by granulations at the lower part.

Twelfth day.—From this period nothing occurred to interrupt the cure of the wound, save an infiltration of matter under the integuments, towards the lower and outer side of the arm. This sinus being laid open, a free and depending opening was afforded.

On the twenty-ninth day the patient was walking about, his arm supported in a sling, and his wound healed.

Thirty-fifth day.—He has regained a slight use of the extensor muscles of the fingers. The numbness and want of feeling are gradually disappearing. Electricity in the course of the brachial nerves and stimulating frictions are now daily employed, and motion given to all parts of the limb.

On the 16th of April the patient was dismissed the hospital, with returning sensation in the back of the hand, and a tolerably free use of the arm.

How far the other nerves supplying the forearm and hand may contribute, by anastomosis as it were, in restoring the perfect use of those parts divested of the influence of the radial nerve, time must prove. Hitherto something of the sort seems to have commenced; and my expectations are the more sanguine, as to this date, June 2d, 1829, the man has not returned to the Naval Hospital.

The tumor, on examination, was found to consist externally of the thickened neurilema, extending from the nerve above the tumor as a capsule to the nerve below. This general tunic was of a lamellated structure, as viewed by the microscope, dense and inelastic, and of the colour of the tunica albuginea of the testis. Under this the nervous fasciculi appeared diverging and intersecting each other like network, to the thickness of half an inch. The rest of the mass consisted internally of a pulpy, striated, greenish-black matter; the striæ running from the circumference towards the centre, and surrounding minute interstitial cells filled with a similar medullary pulp or jelly. A small portion of coagulated blood was very distinct on one side in the firmer substance of the tumor. The centre was occupied by half a teaspoonful of highly fetid coffee-coloured sanies. Condensed cellular membrane surrounded the whole of the tumor, which, at the inner and lower side, was firmly adherent to that part of the brachialis externus removed with it during the operation. The tumor was an oval of two inches and a half in the long diameter, by nearly two inches across. This, with the portions of nerve divided above and below, brought the whole length of the extirpated part to four inches.

*Observations.* That the blow received on the arm fifteen years ago was the primary cause of the chronic inflammation established in the radial nerve, most probably first in its neurilematic theca or capsule, there is little doubt; but that it should have once commenced, and then have lain dormant for the space of nearly thirteen years, is remarkable. This reproductive power I have had occasion to observe not unfrequently, both in the mammæ and testes, arising either from a blow, the action of cold, or some peculiarity of the system at the time. The epididymis I have found to be most speedily affected by this sudden change, particularly in dramdrinkers, and in those addicted to excess in venery. In these cases it appears to me that the sooner an operation is had recourse to, the better. The preparation of this rare and interesting tumor I have presented to the Hunterian Museum belonging to the Royal College of Surgeons of London.—*Edinburgh Med. and Surg. Journal.*

